# Supplementary material for: Correlation of Plasmatic Amyloid Beta Peptides (Aβ-40, Aβ-42) with Myocardial Injury and Inflammatory Biomarkers in Acute Coronary Syndrome
Source: J Clin Med. 2024 Feb 16;13(4):1117. doi: 10.3390/jcm13041117 (PMC10889335; doi:10.3390/jcm13041117)
Supplement: Supplementary file 1 [file jcm-13-01117-s001.zip › jcm-2845056-supplementary.pdf]

Table S1. (Additional analysis was calculated without outliers†). Shows the correlation between our study variables and Aβ peptides (Aβ-42, Aβ-40), Aβ-42/40 ratio in 57 patients with acute coronary syndrome. Data was analyzed using Spearman's correlation test (Additional analysis was calculated without outliers).

**Variable**	**Aβ-42 (pg/ml)**		**Aβ-40 (pg/ml)**		**Aβ-42/40 ratio**	
	Rho	p value	Rho	p value	Rho	p value
Age (years)	0.191	0.155	0.392	0.003	-0.316	0.016
BMI (Kg/m^2)	0.034	0.803	-0.031	0.820	-0.008	0.951
One or more SMuRF	0.156	0.246	0.231	0.084	-0.215	0.108
High sensitivity troponin I (pg/ml)	-0.003	0.981	0.084	0.536	-0.091	0.500
CRP (mg/dl)	-0.006	0.965	0.028	0.848	-0.069	0.636
NT-proBNP	0.289	0.029	0.290	0.029	-0.177	0.187
Albumin (g/dl)	-0.160	0.311	-0.045	0.775	0.105	0.508
Total cholesterol (mg/dl)	-0.078	0.607	0.131	0.386	-0.092	0.542
HDL (mg/dl)	-0.075	0.613	0.101	0.492	-0.086	0.559
LDL (mg/dl)	-0.080	0.589	0.092	0.536	-0.051	0.732
Triglycerides	0.132	0.394	0.092	0.552	-0.028	0.855
AIP	0.138	0.372	0.049	0.751	0.003	0.982
Symptom-to-blood sample‡	-0.050	0.713	-0.167	0.214	0.070	0.606

† Outliers defined by **Aβ-42** <20 pg/ml or >100 pg/ml, **Aβ-40** >400 pg/ml, **Aβ-42/40 ratio** <1.0 and **NT-proBNP** >10,000 pg/ml. ‡Time between symptom onset and blood sample collection.

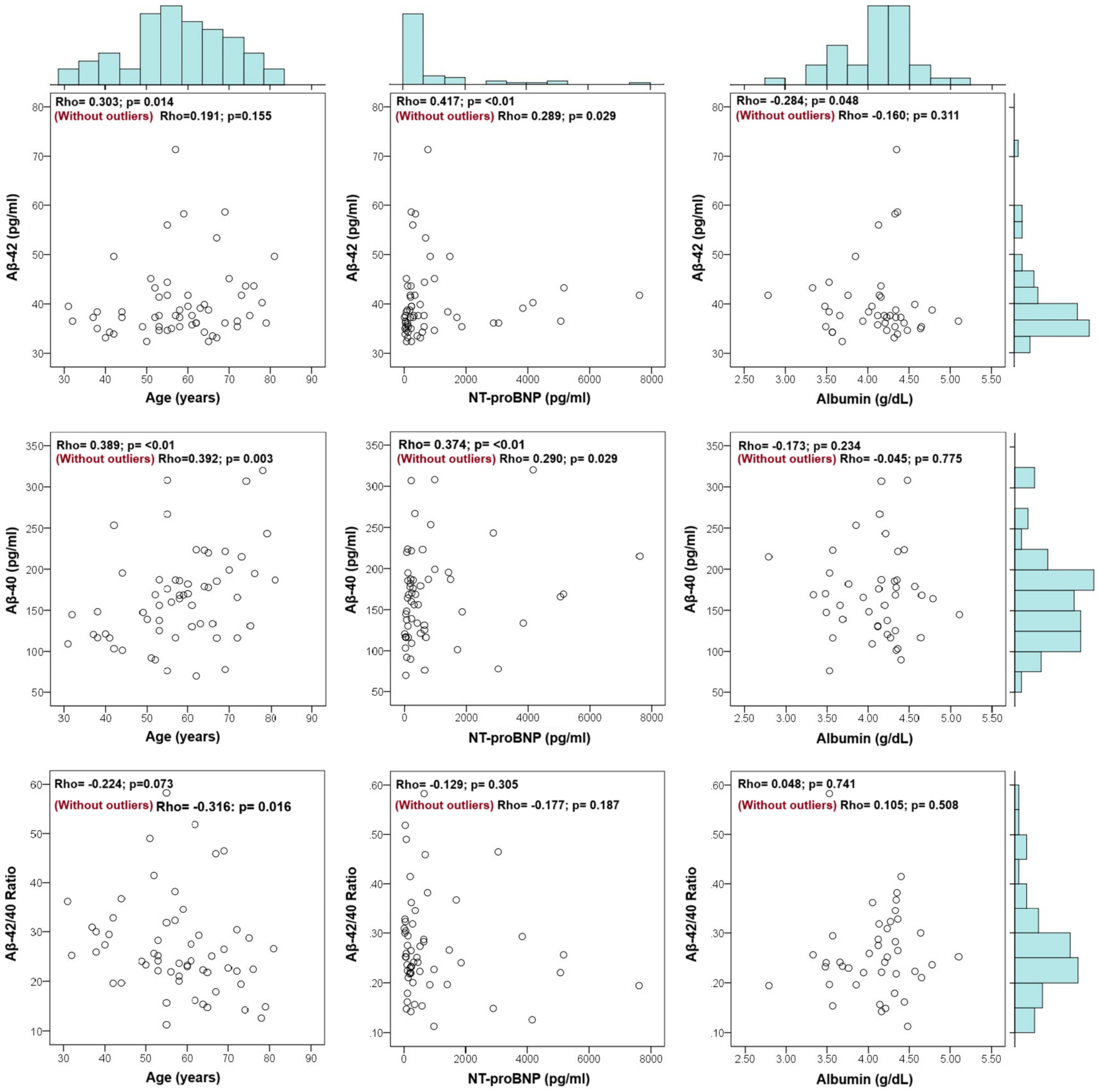

Figure S1. Correlation analysis without outliers. Scatterplot matrix and marginal histograms (with Spearman's correlation coefficients) showing the relation between **Aβ-42** vs age, NT-proBNP, and albumin (*top row*); **Aβ-40** vs age, NT-proBNP, and albumin (*middle row*); **Aβ-42/40 Ratio** vs age, NT-proBNP, and albumin (*bottom row*).

Table S2. (Additional analysis without outliers † was calculated). Demographic characteristics of 57 patients admitted to the coronary care unit grouped by ST elevation myocardial infarction (STEMI) or non-ST elevation myocardial infarction (NSTEMI). Data is shown as mean ± standard deviation, median (percentile 25 – percentile 75), and absolute value (percentage)

Variable	STEMI (N = 30)	NSTEMI (N = 27)	p value
Age (years)	57 ± 13	58 ± 12	0.747
Sex			0.177
Male	27 (87.1%)	20 (74.1%)	
Female	4 (12.9%)	7 (25.9%)	
BMI (Kg/m^2)	28.35 ± 4.2	28.12 ± 3.78	0.826
Overweight	13 (41.9%)	12 (44.4%)	
Obesity	11 (35.5%)	9 (33.3%)	
Overweight/Obesity	24 (77.4%)	21 (77.7%)	
Diabetes	3 (9.7%)	7 (25.9%)	0.099
Hypertension	15 (48.4%)	20 (74.1%)	0.046
Dyslipidemia	6 (19.4%)	6 (22.2%)	0.788
Previous myocardial infarction	7 (22.6%)	15 (55.6%)	0.010
Smoking status			0.005
Current smoker	15 (48.4%)	5 (18.5%)	
Former smoker	5 (16.1%)	15 (55.6%)	
Nonsmoker	11 (35.5%)	7 (25.9%)	
Number of SMuRFs			0.122
One or more	25 (83.3%)	26 (96.3%)	
None	5 (16.7%)	1 (3.7%)	
NYHA			0.404
≥2 Class	4 (12.9%)	2 (7.4%)	
Killip-Kimball			0.138
≥2 Class	8 (25.8%)	3 (11.1%)	
GRACE			0.658
Intermediate-High Risk	19 (61.3%)	15 (55.5%)	
TIMI			0.501
Intermediate-High Risk	18 (58.1%)	18 (66.7%)	
CRUSADE			0.681
Moderate-High Risk	11 (35.5%)	11 (40.7%)	
LVEF			0.373
Mid-range ejection fraction	11 (36.7%)	7 (28.0%)	
Reduced ejection fraction	7 (23.3%)	5 (20.0%)	
Symptom-to-door time (minutes)	383 ± 193	284 ± 171	0.045
Symptom-to-blood sample time ‡ (minutes)	409 ± 197	366 ± 182	0.391
Door-to-ECG time (minutes)	6 (5-8)	5 (5-7)	0.113
Door-to-balloon time (minutes)	508 (95-2394)	2193 (1179-4154)	0.003
Symptom-to-catheter time (minutes)	1763 ± 1973	3180 ± 2612	0.049

† Outliers defined by **Aβ-42** <20 pg/ml or >100 pg/ml, **Aβ-40** >400 pg/ml, **Aβ-42/40 ratio** <1.0 and **NT-proBNP** >10,000 pg/ml. ‡Time between symptom onset and blood sample collection

Table S3. Biochemical values from 65 patients admitted to the coronary care unit grouped by ST elevation myocardial infarction (STEMI) or non-ST elevation myocardial infarction (NSTEMI). Data is shown as mean ± standard deviation or median (percentile 25 – percentile 75)

Variable	STEMI (N = 30)	NSTEMI (N = 27)	p value
Aβ-42 (pg/ml)	35.96 (34.27-39.64)	41.76 (38.99-47.75)	0.001
Aβ-40 (pg/ml)	169.38 ± 88.26	183.68 ± 68.96	0.472
Aβ-42/40 ratio	0.25 (0.21-0.30)	0.24 (0.20-0.38)	0.708

Table S4. (Additional analysis without outliers†) Biochemical values from 57 patients admitted to the coronary care unit grouped by ST elevation myocardial infarction (STEMI) or non-ST elevation myocardial infarction (NSTEMI). Data is shown as mean ± standard deviation or median (percentile 25 – percentile 75)

**Variable**	**STEMI (N = 30)**	**NSTEMI (N = 27)**	**p value**
Aβ-42 (pg/ml)	35.39 (34.27-37.64)	40.26 (37.64-45.13)	0.001
Aβ-40 (pg/ml)	159.62 ± 49.59	172.26 ± 63.56	0.399
Aβ-42/40 ratio	0.24 ± 0.07	0.28 ± 0.12	0.118
High sensitivity troponin I (pg/ml)	354.0 (67.10-4742.0)	247.0 (65.3-1021.0)	0.315
NT-proBNP (pg/ml)	231.0 (74.6-642.0)	315.0 (194.0-978.0)	0.161
CRP (mg/dl)	4.99 (2.65-9.41)	3.84 (0.99-7.52)	0.198
Albumin (g/dl)	4.14 ± 0.43	4.06 ± 0.46	0.540
Total cholesterol (mg/dl)	172.5 ± 40.9	163.6 ± 32.7	0.434
Triglycerides (mg/dl)	165.5 ± 65.4	162.9 ± 55.0	0.889
HDL (mg/dl)	38.5 ± 9.1	35.7 ± 8.0	0.273
LDL (mg/dl)	114.7 ± 39.2	102.1 ± 30.8	0.227
AIP	0.25 ± 0.22	0.29 ± 0.15	0.472

† Outliers defined by **Aβ-42** <20 pg/ml or >100 pg/ml, **Aβ-40** >400 pg/ml, **Aβ-42/40 ratio** <1.0 and **NT-proBNP** >10,000 pg/ml.
